# Patient-derived outcome assessment of knowledge, communication, and management in those diagnosed with *BAP1*-tumor predisposition syndrome

**DOI:** 10.1007/s10689-026-00541-8

**Published:** 2026-03-17

**Authors:** Kelley Godwin, Joseph McElroy, Leigha Senter, Lindsey Byrne, Mohamed H. Abdel-Rahman

**Affiliations:** 1 Present Address: Division of Human Genetics, Department of Internal Medicine, The Ohio State University Wexner Medical Center, Columbus, OH USA; 2 Department of Biomedical Informatics, Center for Biostatistics, College of Medicine, The Ohio State University, Columbus, OH USA; 3 Present Address: Department of Ophthalmology and Visual Sciences, The Ohio State University, Columbus, OH USA

**Keywords:** BAP1, BAP1-TPDS, Hereditary, Neoplastic syndromes, Patient-reported outcomes, Health knowledge

## Abstract

Germline pathogenic variants in *BAP1* are associated with *BAP1* tumor predisposition syndrome (*BAP1*-TPDS), and an increased risk for different cancers and preneoplastic lesions. This study assessed the degrees of patient knowledge of *BAP1*-TPDS, patient-reported sharing of their genetic test results and diagnosis, and the degree of compliance with current management guidelines. A survey was sent to individuals with a germline pathogenic/likely pathogenic variant in *BAP1* who are enrolled in a research registry at The Ohio State University. Knowledge about *BAP1*-TPDS was assessed utilizing a modified scale (KnowGene). Participants were asked about communication surrounding *BAP1*-TPDS with family members, and their current cancer surveillance. Forty-two (55%) subjects completed the survey with 25 (60%) having strong 80% knowledge of the syndrome, and 33 (79%) having at least 60% knowledge. All participants reported sharing their genetic result or diagnosis with at least one family member, primarily their first-degree relatives. Most of the cohort had undergone some recommended surveillance for *BAP1*-TPDS. In conclusion, *BAP1*-TPDS subjects are knowledgeable about the syndrome phenotype, highly communicative about their diagnosis with family members, and follow some recommended surveillance. Capturing patient-reported outcomes of *BAP1*-TPDS subjects is crucial for understanding their need for further education, resources, or support.

**Supplementary Information:**

The online version contains supplementary material available at 10.1007/s10689-026-00541-8.

## Introduction

Germline pathogenic variants in the *BAP1* (BRCA-Associated Protein 1) gene are associated with *BAP1* tumor predisposition syndrome (*BAP1*-TPDS) and are associated with an increased risk for the development of four main cancers: uveal melanoma (UM), cutaneous melanoma (CM), malignant mesothelioma (MMe), and renal cell carcinoma (RCC) [1–3]. *BAP1*-TPDS can also be associated with preneoplastic melanocytic tumors, referred to as *BAP1*-inactivated melanocytic tumors (BIMTs) [4]. There is emerging evidence for associations with basal cell carcinoma (BCC), meningioma, cholangiocarcinoma, and hepatocellular carcinoma [1, 5–12]. Since *BAP1*-TPDS is a more recently described hereditary cancer syndrome, phenotypic associations continue to evolve.

There are guidelines for risk management for RCC in those with germline pathogenic or likely pathogenic (P/LPV) variants in *BAP1* published by the National Comprehensive Cancer Network® (NCCN®) [13]. Since 2020, it has been recommended that those with *BAP1*-TPDS obtain an abdominal MRI or CT (MRI preferred), obtained with and without IV contrast, every 2 years starting at age 30 or 10 years earlier than the earliest age of diagnosis in the family [13]. Our group has helped create consensus guidelines, along with others who have developed additional recommended guidelines for UM, CM, and MMe [14–17]. For UM, this includes annual dilated eye exams and baseline ophthalmologic imaging/dilated fundus imaging, with referral to an ophthalmologist who specializes in management of UM for any suspicious lesion [14–17]. Some have recommended these exams begin at age 11 years, while others have recommended they begin at age 16 years, with recommendation to increase the frequency to twice a year beginning at age 30 years [14–16]. For CM, annual full-body skin examination by a dermatologist specialized in melanoma is recommended beginning around age 18 years, with consideration for total body photography in those with a large number of lesions [14, 15, 17]. For MMe, there is no consensus on screening modalities, though the recommendation is to undergo biannual abdominal MRI combined with RCC evaluation beginning at age 30 years, and for any clinical manifestations of MMe to be referred to oncologist with expertise in the management of MMe [14, 15, 17].

Patient-reported outcomes have been published in cohorts of individuals diagnosed with Hereditary Breast and Ovarian cancer (HBOC) syndrome and Lynch syndrome [18–21]. Since there are no published patient-reported outcomes specific to those diagnosed with *BAP1*-TPDS, this study provides the first insight. For individuals with *BAP1* germline P/LPV, we assessed participants’ knowledge of the *BAP1*-TPDS cancer phenotype, extent of sharing diagnoses with family, and the adherence to *BAP1*-TPDS recommended cancer surveillance. We also evaluated involvement in support groups, experiences with genetic counselors, whether they had to educate their healthcare providers about *BAP1*-TPDS, and if there were discussions about *BAP1*-related risks with providers.

## Materials and methods

### Study design

In collaboration with the Center for Clinical and Translational Science (CCTS) at The Ohio State University (OSU), College of Medicine, we developed a 46-question survey utilizing REDCap software. The survey contained both single and multi-part questions, as well as skip-logic. The survey was distributed via email or administered via telephone between late September 2023 through early November 2023 to individuals with P/LPV in *BAP1* who had enrolled in the Frequency and Clinical Phenotype of *BAP1* Hereditary Predisposition Syndrome Study at The Ohio State University (hereto forth also referred to as: *BAP1* Patient Registry; the study is listed at clinicaltrials.gov: NCT04792463). There was no incentive, monetary or otherwise, to participate in the survey.

### Eligibility criteria

Participants were eligible if they were enrolled in the *BAP1* Patient Registry, were positive for a germline P/LPV in *BAP1*, provided contact information, and were aged 18 years or older. There were 196 individuals enrolled in the *BAP1* patient registry, with 77 that were eligible. For the remaining subjects five were deceased, 26 had variants of uncertain significance and 88 tested negative for the *BAP1* family variants.

Demographics were collected on those who were eligible: age, biological sex, self-reported ancestry, highest year of school or degree completed, work status, and annual household income. We also collected whether the participants had a history of a cancer diagnosis, whether participants were the proband of their family or what their relationship to the proband is, and whether participants responded to the survey by email or phone.

### Knowledge

Investigator-developed questions were created and based on a knowledge scale, KnowGene [22, 23]. Correct answers were pre-determined by current published knowledge and clinical practice. Knowledge was measured by assessing the number of correct responses provided for each of five items. Four items for assessing participant’s knowledge about *BAP1*-TPDS were adapted from KnowGene for evaluating general cancer genetic knowledge relevant to multigene panel testing [22, 23]. Responses were presented on 5-point Likert scales: Strongly Disagree, Disagree, Neither Agree nor Disagree, Agree, Strongly Agree, or Do Not Know. The fifth item listed multiple types of cancers/tumors in patient-friendly terms and asked the participant to select all that are associated with *BAP1*-TPDS.

### Family communication and sharing results

Participant’s sense of responsibility to share results with family members was measured with two items. Participants were asked if someone with *BAP1*-TPDS should share their genetic results with family members who could be at risk, and if they feel a responsibility to share their diagnosis with family members. Responses were presented on 5-point Likert scales. Additionally, participants were asked if they had told at least one family member about their *BAP1* result. Skip logic was utilized so that only those who reported some amount of family sharing were asked to describe with which family members they shared information, expanding out to four generations, and an option for an open-ended textbox to explain relatives otherwise not listed.

### Exploratory assessments

Additional assessments covered a variety of topics related to *BAP1*-TPDS. A full list of questions is available in Table S-1.

Further assessment into participants’ discussion with their healthcare provider about *BAP1*-TPDS included asking whether participants had to educate their healthcare provider through explaining *BAP1*-related cancer risks and/or screening recommendations. We also asked participants how much discussion they felt they had with their healthcare provider about *BAP1*-related risk topics, such as the risk of cancer for family members, the risk of developing skin, eye, and kidney cancers, mesothelioma, and the risk of developing other cancer types with a text box to specify further. For the amount of discussion, participants could select: Not at all, Some, A lot, Do not remember, or Prefer not to answer.

To evaluate how participants follow the recommended surveillance for *BAP1*-TPDS based on both NCCN Guidelines® and consensus screening guidelines, we asked about the following: Physical exam by primary care provider, Skin exam by dermatologist, Exam of the back of the eye after dilatation by ophthalmologist or optometrist, Photos of the back of the eye, Ultrasound of the abdomen, MRI of the abdomen, and Other (with a text box to specify type of surveillance) [13–17]. For each of these screenings, the frequency options participants could select were: I have never had this screening, Every 6 months, Yearly, Every 2 Years, Do not know, Prefer not to answer, or Other (with a text box to specify surveillance frequency).

### Data analysis

Descriptive statistics were used to quantify participant characteristics and responses across the survey into groups.

Knowledge was measured by five items and scored as correct/incorrect. For calculation purposes, if the correct answer was an agreement with the statement, both Agree or Strongly Agree responses were counted as correct; if the correct answer was a disagreement with the statement, both Disagree or Strongly Disagree responses were counted as correct. All other answers to these questions were considered not the correct answer. Correct answers were given a score of 1 and not correct answers were given a score of zero. Correct answers were totaled and divided by the number of items (n = 5) to create a “Knowledge Score” percentage for each participant. If a participant opted out of answering these questions by selecting “Do Not Know,” the response was considered incorrect for analysis purposes. Knowledge Scores were also compared to participant characteristics, such as age, biological sex, whether the participants had a history of a cancer diagnosis or not, and whether participants responded to the survey by email or phone via generalized linear models with Knowledge Score as the dependent variable.

For the item linking which cancers are associated with *BAP1*-TPDS, each of the four correct answers (for the four known cancer types: eye cancer for UM, skin cancer for CM, mesothelioma for MMe, and kidney cancer for RCC) were analyzed as one, meaning that all four of these cancer types had to be selected by a participant in order to be considered correct. If someone selected more than the four cancers, the additional selections were counted as not correct. Data shows there are other cancer types associated with *BAP1*, but these four cancers have the strongest data associations. Table 1 includes the knowledge questions, the response options, and the correct answers used for scoring.

**Table 1:** Questions, response options, and correct answers for contribution to Knowledge Score of BAP1-TPDS

Item to assess knowledge	Response options	Correct Answer(s)
People with *BAP1*-TPDS have a higher chance to develop more than one type of cancer compared to others	Strongly disagree, disagree, neither agree nor disagree, agree, strongly agree, do not know	Agree, strongly agree
A person with *BAP1*-TPDS will definitely get cancer 1 day	Strongly disagree, disagree, neither agree nor disagree, agree, strongly agree, do not know	Strongly disagree, disagree
People with *BAP1*-TPDS may get cancer at a younger age than people with average risk cancer	Strongly disagree, disagree, neither agree nor disagree, agree, strongly agree, do not know	Agree, strongly agree
*BAP1*-TPDS is hereditary (runs in the family)	Strongly disagree, disagree, neither agree nor disagree, agree, strongly agree, do not know	Agree, strongly agree
Of the following cancers/tumors, which to your knowledge are associated with *BAP1*-TPDS? Check all that apply:	Prostate cancer Skin cancer Eye cancer Kidney cancer Mesothelioma Lung cancer Ovarian cancer Breast cancer Pancreatic cancer Meningioma tumors Liver cancer Bile duct cancer	Skin cancer, eye cancer, kidney cancer, and mesothelioma

## Results

### Sample demographics

Of the 77 eligible individuals, 42 (55%) participated in the survey. Thirty-five (45%) individuals were unable to be reached through multiple attempts. Most participants were female, 50 years old or older, and mostly White European (Table 2). Of those who did not participate, nineteen (54%) were female (data not shown). No other demographic data was available for non-participants. Demographic data did not show any meaningful trends or comparisons with any other variable in the survey.

**Table 2:** Participants’ demographic data

Participant demographics	N = 42 n (%)
*Age (years)*	
20–29	3 (7.1%)
30–39	6 (14.3%)
40–49	6 (14.3%)
50–59	8 (19.0%)
60–69	13 (31.0%)
70–79	5 (11.9%)
80–89	1 (2.4%)
*Biological sex*	
Male	15 (35.7%)
Female	27 (64.3%)
*Ancestry (could select more than one)*^a^	
Black or African American	2 (4.8%)
Hispanic, Latino/a/x, or Spanish origin	1 (2.4%)
White or European	39 (92.9%)
*Highest year of school or degree completed*	
Grades 9–11 (Some high school)	1 (2.4%)
Grade 12 or GED (High school diploma)	5 (11.9%)
Trade, technical, or vocational school certification/degree	2 (4.8%)
Associate’s degree	4 (9.5%)
Bachelor’s degree	14 (33.3%)
Master’s degree or higher	15 (35.7%)
Prefer not to answer	1 (2.4%)
*Work status*	
Full-time	22 (53.7%)
Not full-time	19 (46.3%)
*Annual household income from all sources*	
< $100,000	14 (33.3%)
$100,000 to < $200,000	15 (35.7%)
> $200,000	6 (14.3%)
Prefer not to answer	7 (16.7%)

^a^Other ancestry options presented were not selected

**Fig. 1:**
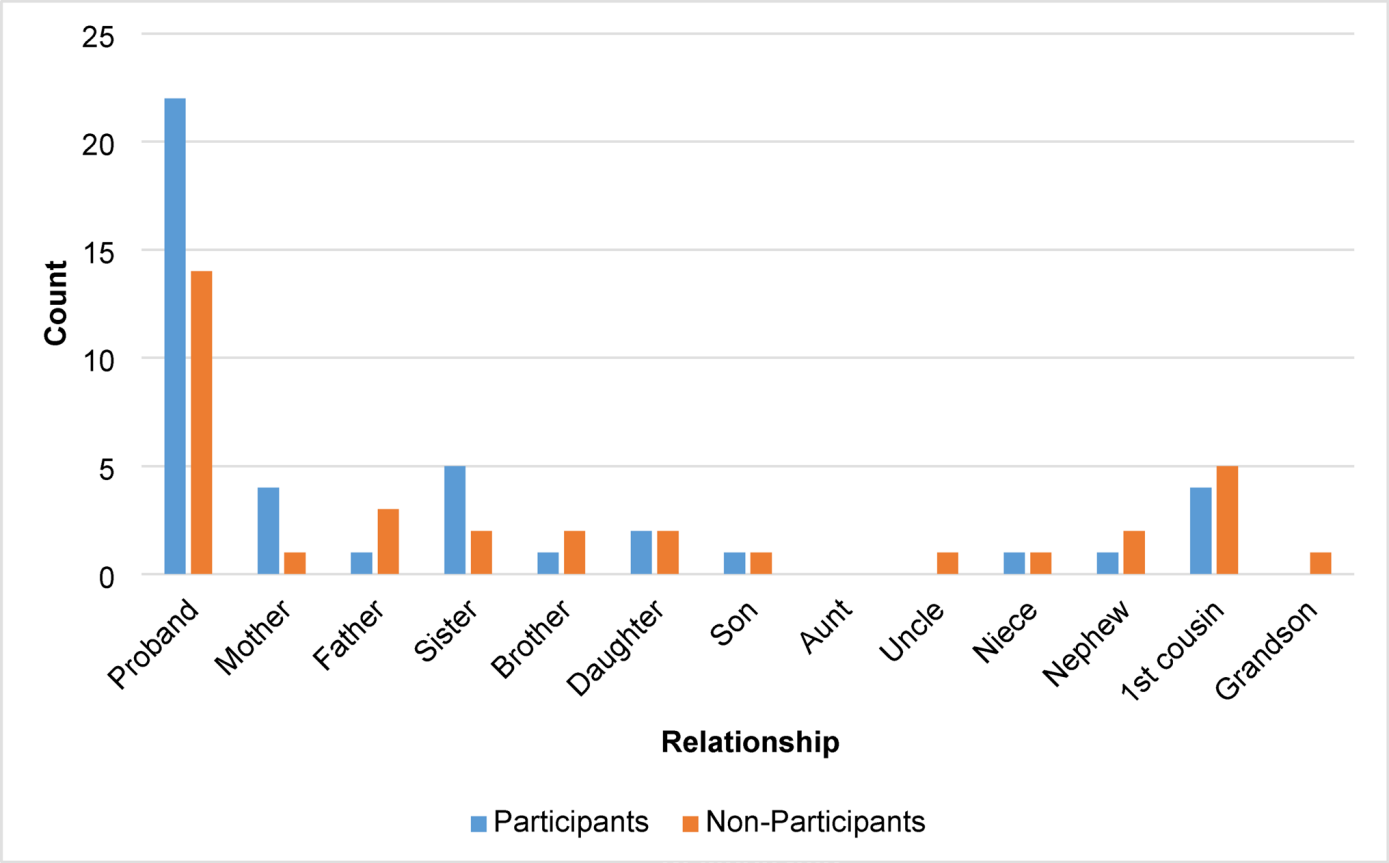
Distribution of proband or non-proband relationships classified among participants and non-participants

Among survey participants, twenty-two (52%) were probands, and other survey participants mostly identified as first-degree relatives (Fig. 1).

Participant and non-participant information is detailed in Table S-2. In both participant and non-participant groups, more individuals had a personal history of a cancer diagnosis than not: 29 (69%) participants compared to eighteen (51%) non-participants. Nine (21%) participants had a history of RCC compared to two (6%) non-participants. Also notable was that six (14%) participants had a history of UM, and zero (0%) non-participants had this history. There were four individuals in each group who were probands with no personal history of cancer. Among them, three (7%) participants and 2 (6%) non-participants had BIMTs.

### Knowledge

Knowledge scores were calculated for each participant to determine knowledge surrounding *BAP1*-TPDS. Although no participant scored 100% for knowledge about *BAP1*-TPDS, 25 (59.5%) had a high score of 80%, meaning that they responded correctly to four out of five of the assessment items (Table 1). Next frequent score was 60%, with eight (19%) who had responded correctly to three out of five of the assessment items. The assessment item, “A person with *BAP1*-TPDS will definitely get cancer 1 day,” had responses with the most variety: 22 (52%) of participants responded correctly with either Disagree or Strongly Disagree, six (14%) responded incorrectly with either Agree or Strongly Agree, and the remaining responded incorrectly with eleven (26%) who responded Neither Agree nor Disagree or three (7%) who responded Do Not Know. The assessment item, “*BAP1*-TPDS is hereditary (runs in the family),” had the most correct responses, to which 41 (98%) participants responded correctly with either Agree or Strongly Agree, and only one (2%) responded incorrectly with either Disagree or Strongly Disagree. Analyses to identify if variation in knowledge was associated with whether the participant was a proband or non-proband, or whether the participant had a positive or negative history of cancer, revealed no statistical significance.

**Fig. 2:**
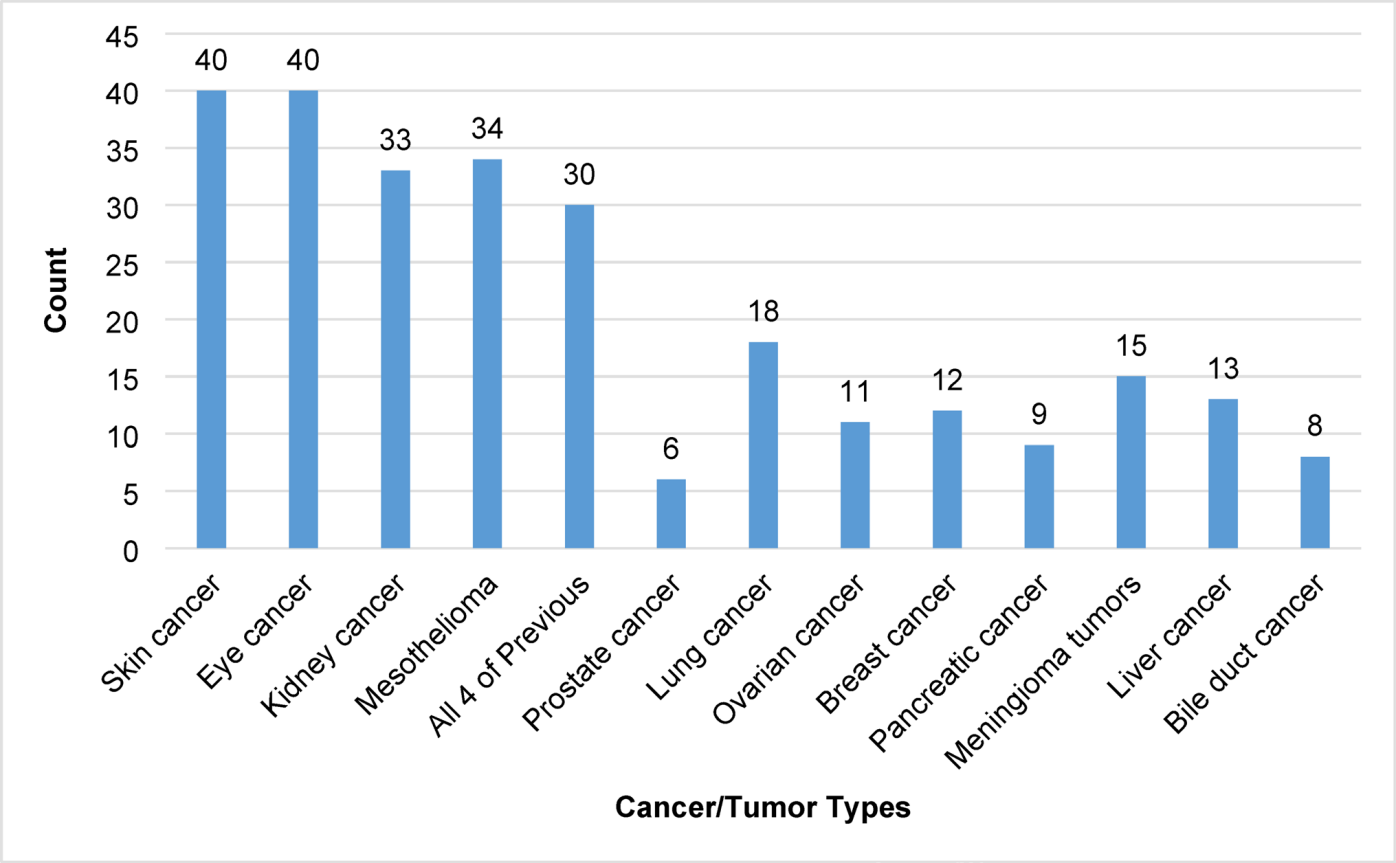
Participant-reported cancer and tumor types perceived to be associated with *BAP1*-TPDS

To further assess their knowledge of the *BAP1*-TPDS phenotype, participants selected all associated known cancers and tumors from a list (Fig. 2). Most participants selected the known cancer types: 40 (95%) selected skin cancer (CM), 40 (95%) selected eye cancer (UM), 34 (81%) selected mesothelioma (MMe), and 33 (79%) selected kidney cancer (RCC). Moreover, 30 (71%) participants identified all four main cancers associated with *BAP1*-TPDS, though no participant selected only these four cancer types, and three (7%) participants selected every cancer type/tumor listed.

### Family communication and sharing

**Fig. 3:**
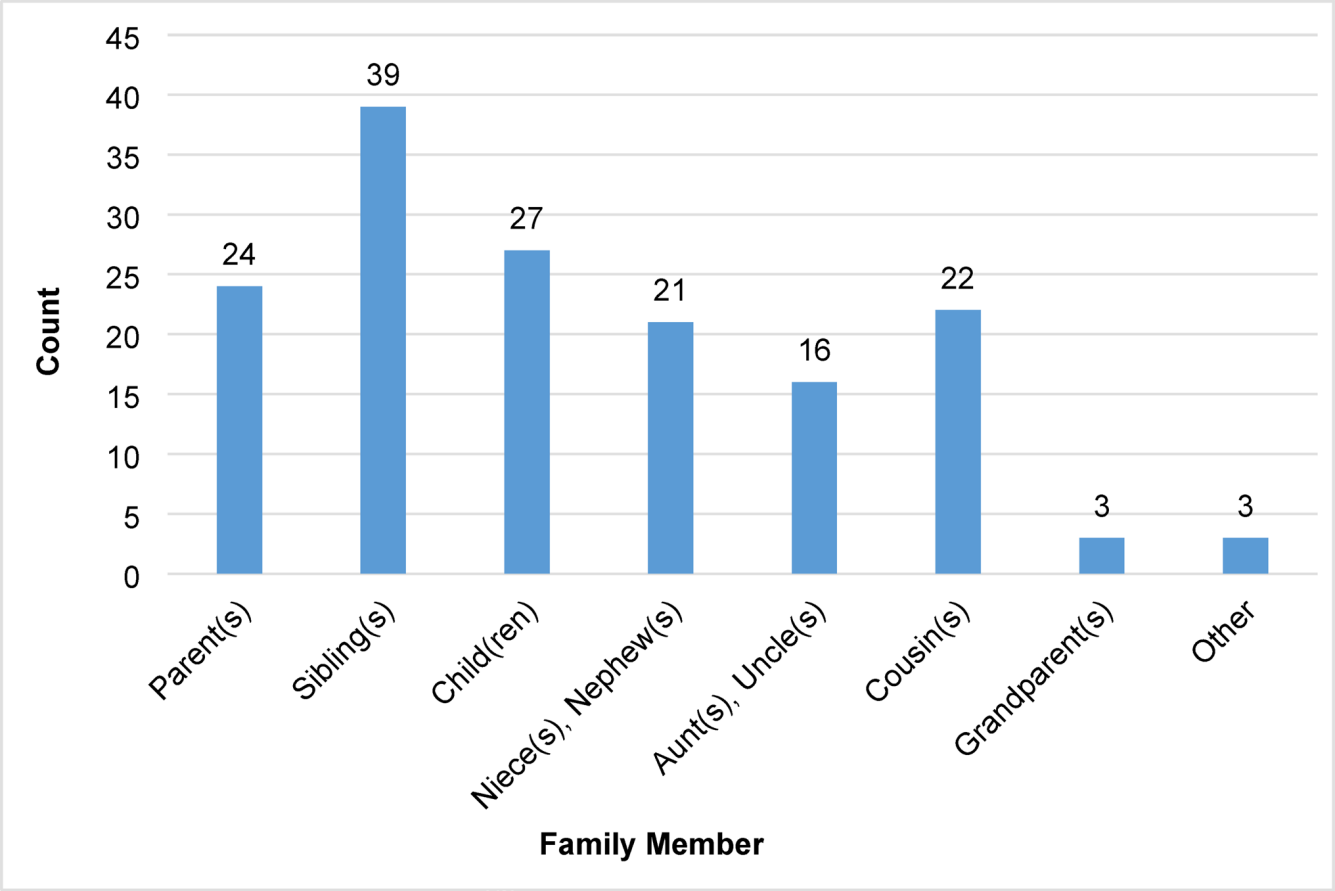
Participant-reported sharing of BAP1-TPDS information across family members

Participants overwhelmingly felt a sense of responsibility to share their *BAP1* status with family members: 27 (64%) strongly agreed and thirteen (31%) agreed with feeling that responsibility. All 42 (100%) participants responded that they had told at least one family member about their *BAP1* genetic test result. Thirty-nine (93%) participants reported sharing information about *BAP1*-TPDS with their sibling(s), followed by 27 (64%) sharing with their child(ren), and 24 (57%) sharing with their parent(s) (Fig. 3).

### Exploratory assessments

#### Discussion about cancer risks and educating healthcare providers

**Fig. 4:**
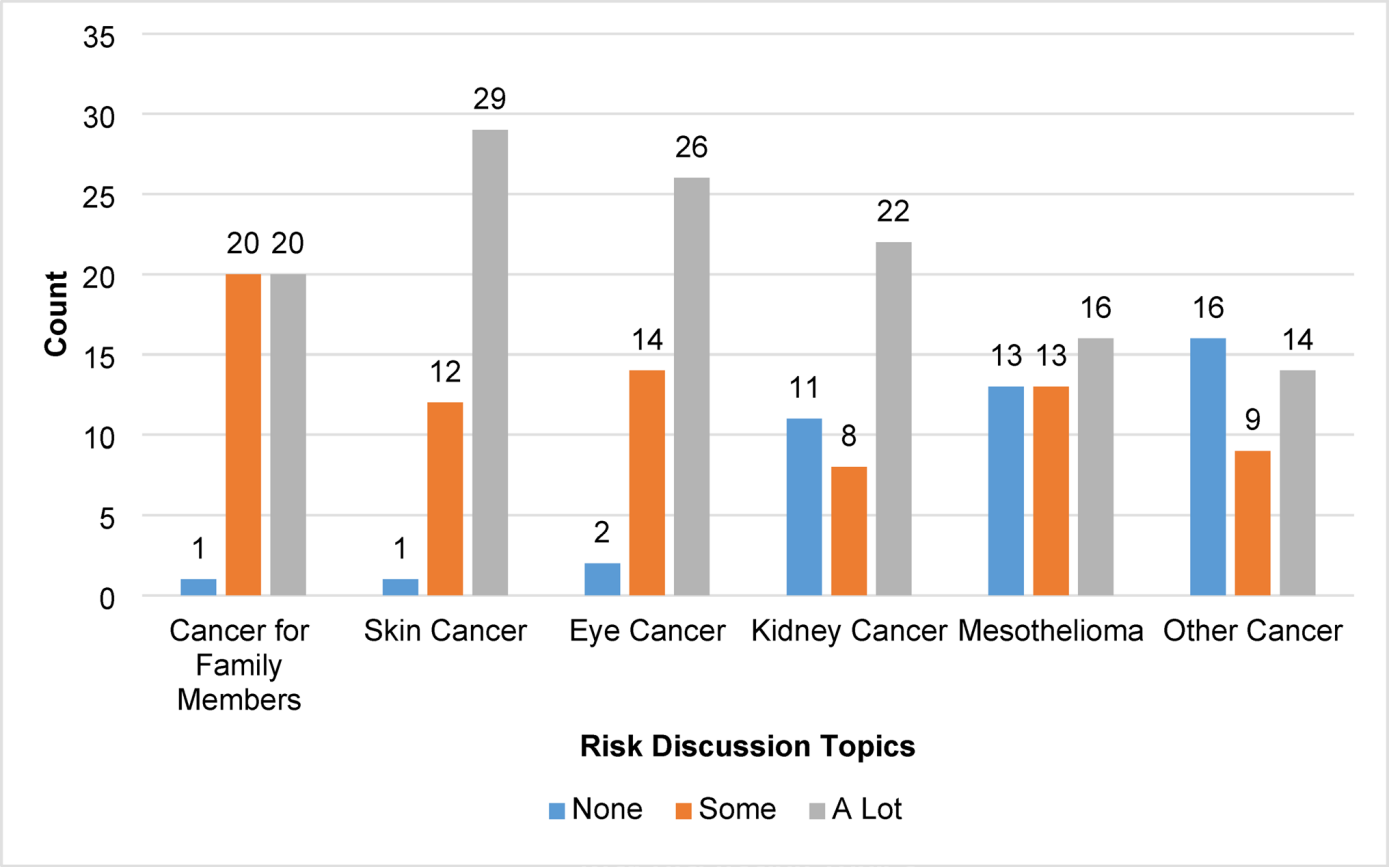
Participant-reported level of discussion with healthcare providers about *BAP1*-TPDS risk topics

Twenty-two (52%) participants reported that they had to educate their healthcare provider about *BAP1*, such as explaining cancer risks and/or screening recommendations, while sixteen (38%) reported they did not have to do this, three (7%) reported they did not remember, and one (2%) individual preferred to not answer. Additionally, participants were asked how much discussion they had with their healthcare provider about various risk topics related to *BAP1*-TPDS. Twenty-nine (69%) participants reported a lot of discussion about skin cancer risk, followed by eye cancer (62%), kidney cancer (52%), and family cancer risk (48%) (Fig. 4). Thirteen (31%) participants reported never having discussed the risk of developing mesothelioma.

#### Cancer surveillance

Lastly, patients were asked about the frequency of which they undergo the recommended cancer screenings for *BAP1*-TPDS. Most of the participants undergo annual screenings: 24 (57%) undergo annual skin examination, 33 (79%) undergo a physical examination, 27 (64%) undergo examination of the back of the eye after dilatation, and 23 (55%) have photos taken of the back of the eye. The types and frequency of kidney imaging varied. Twenty (48%) participants reported never having undergone an ultrasound of the abdomen and eleven (26%) participants never having undergone an MRI of the abdomen. More participants reported having annual abdominal imaging than biannual—with thirteen (31%) participants reported they undergo annual MRI of the abdomen compared to the recommended biannual MRI, reported by seven (17%) participants; nine (21%) participants reported annual ultrasound of the abdomen compared to five (12%) who reported biannual ultrasound. At the time of our survey, two participants were under the age of 30 years and enrolled in the *BAP1* Patient Registry in 2019; both reported having no history for imaging of the kidney, which is consistent with established guidelines [13]. One participant under age 30 years at enrollment in the *BAP1* Patient Registry in 2019 reported annual abdominal MRI. Another participant, under age 30 at enrollment in 2018 but over 30 at the time of the survey, reported annual abdominal ultrasound but no MRI. Some participants reported additional *BAP1*-TPDS cancer screenings: eight (19%) every 6 months, thirteen (31%) yearly, two (5%) every 2 years, and ten (24%) reported none. Three (7%) were unsure, two (5%) preferred not to answer, and four (10%) reported other frequencies. Due to variability, these “other” screenings were not further analyzed.

## Discussion

This study is the first to assess patient-reported outcomes in the *BAP1*-TPDS population, with a notably high response rate of 54%. Additionally, this study demonstrates that our cohort is knowledgeable, and highly communicative about their diagnosis and genetic test results with all family members.

### Knowledge

Overall, participants had high levels of knowledge related to cancer risk and inheritance of *BAP1*-TPDS. Similar knowledge scores have been reported in the HBOC population, with an average of 40–70% on a knowledge scale related to the genetics and risks of HBOC [24]. Another group reported moderate knowledge among individuals with ovarian cancer, with a mean score of 11.9/19, or about 63%, though they reported fewer than half of the participants correctly answered items related to inheritance, clinical impact, and interpretation of genetic test results [25].

Our patient cohort had similar knowledge of cancers associated with *BAP1*-TPDS as providers who were familiar with the syndrome. In a conference abstract, medical providers familiar with *BAP1*-TPDS were surveyed about *BAP1*-associated cancers and surveillance and found providers were knowledgeable of the four main cancers: UM (94%), CM (90%), MMe (84%), and RCC (88%). These providers also reported that other cancers were associated with basal cell carcinoma (47%), cholangiocarcinoma (26%), and liver cancer (10%), which do not have a confirmed association with *BAP1*-TPDS at this time (unpublished data). Future studies comparing the knowledge of this cohort about *BAP1*-TPDS with that of the medical professionals who care for them would be valuable.

### Family communication and sharing

Our cohort overwhelmingly agreed/strongly agreed that a person with *BAP1*-TPDS should share their genetic test results with family members. Additionally, they felt a sense of responsibility to share their *BAP1* result with family members. All participants reported sharing their *BAP1* genetic test result or *BAP1*-TPDS diagnosis with at least one family member. About half of participants in this cohort told at least one cousin about their diagnosis, which is similar to what has been reported in Lynch syndrome, with 70% of individuals diagnosed with Lynch syndrome told at least one cousin about their genetic test results [26].

### Management

Most of our cohort reported having annual physical examinations, skin examinations, eye examinations, and photos taken of the eye. There is considerable variety in types and frequency of RCC surveillance, as many participants reported never having undergone imaging of the abdomen. We asked about both abdominal MRI and ultrasound to better understand participants’ imaging practices for RCC surveillance, given the relatively recent introduction of NCCN Guidelines® in 2020 [13]. Of those who did have imaging of the abdomen, more participants reported undergoing this screening annually than the recommended biannual imaging, and more participants reported utilizing MRI than ultrasound. Of note, we did not confirm the reported imaging according to the participant with medical records, and it is possible participants may not know the difference between MRI and ultrasound. Additionally, for many of our participants, their screening guidelines have likely changed over time, and we do not know what they were initially told nor how frequently they are updated, which presents a challenge in fully understanding what participants have been recommended for surveillance protocols. Management decisions are complex, and other factors likely influence participants’ cancer screenings and their frequency. Following recommended cancer screenings in other hereditary cancer syndromes, such as HBOC and Lynch syndrome, also falls short of 100%. Following through with certain recommendations may depend on access to specialized care centers and/or dedicated hereditary cancer screening programs [27–29].

Many individuals in this cohort reported having to educate their healthcare provider about *BAP1*-TPDS, which may include cancer risks and screening guidelines. Since *BAP1*-TPDS is a newer hereditary cancer syndrome and less widely recognized than HBOC or Lynch syndrome, it is understandable that some healthcare providers may be unfamiliar with it. The NCCN Guidelines® only added RCC screening recommendations for *BAP1*-TPDS in 2020, which means there was a lack of established screening guidelines for healthcare providers to follow prior to this time [13]. Twenty-six (62%) of our participants had genetic testing and were enrolled in the *BAP1* Patient Registry prior to 2020. Our data was collected in 2023, which leaves the possibility that some of the cohort may have not had their screening plan updated at the time of survey distribution.

### Discussion about cancer risks

There was variability in the level of discussion participants reported having with their healthcare provider about cancer risks associated with *BAP1*-TPDS. Interestingly, most participants reported having a lot of discussion about the risk for CM and UM, which is likely because these cancers were the first recognized as part of the syndrome phenotype [1]. Multiple participants reported having no discussion with their healthcare provider about the risks for RCC. While there are established NCCN Guidelines® for kidney cancer, explanations for this may be that the screening guidelines are new as of 2020, and that the frequency of RCC is much lower than the frequencies of other cancers reported in *BAP1*-TPDS [12, 14]. The same trend was noted for discussion about the risk for MMe, in which almost one-third of participants reported having no discussion with their healthcare provider. However, there are no consensus on screening guidelines for MMe at this time, though some have suggested an additional evaluation of the peritoneum or pleura during an abdominal MRI for screening of RCC [14, 15, 17]. Others have suggested screening for MMe should be offered only in a research setting [16]. This revealed notable gaps in participants’ reported discussions about cancer risks. Additional chart reviews could clarify whether screenings are occurring despite the reported absence of risk discussions with healthcare providers.

### Study limitations

This study has several limitations. The primary limitation is the selection bias in utilizing a *BAP1* patient natural history registry, in which the enrolled individuals were likely highly motivated to participate. It is important to consider variables such as socioeconomic, education and health statuses that may affect *BAP1* registry participants’ motivation or ability to respond to follow-up questionnaires. To avoid this selection bias in the future, employing other routes for deploying a survey would help to expand and diversify the study cohort.

Another limitation of this study was the lack of diversity in our cohort. Nearly all the participants identified as White or of European background. However, this is not an inherent bias with this survey, as participants on the Frequency and Clinical Phenotype of *BAP1* Hereditary Predisposition Syndrome Study also predominantly identify as White or of European background.

Lastly, the modest sample size reduced the power of the statistical tests for associations with Knowledge Score and limits confidence in the estimates from these models. These patient-reported outcomes may not be comprehensive of the entire *BAP1*-TPDS population, but rather just the individuals in this study cohort who have likely been provided similar education and counseling.

### Directions for future studies

In the current study, we found that the most frequent cancers among survey respondents included basal cell carcinoma, breast cancer, and renal cell carcinoma. However, the existing literature tends to report mesothelioma and uveal melanoma as the most common phenotypes of this syndrome [12]. A potential explanation of these differences is ascertainment bias associated with studies focusing on patients/families with strong histories of one or more cancers. Our registry was open to all subjects with germline *BAP1* variants irrespective of the cancer phenotype, so it is likely more representative of the spectrum of the *BAP1*-TPDS than studies focusing on one cancer phenotype. Other explanations are that several subjects with mesothelioma and uveal melanoma were deceased at the time of study, and that many of our uveal melanoma patients with germline *BAP1* P/LP variants were enrolled in a separate protocol to study the genetics of uveal melanoma and were not included. Conducting chart reviews on participants would be valuable as a future study. Chart reviews can provide more accurate insight into what *BAP1*-TPDS surveillance participants are undergoing, including specific imaging types and frequencies. Another future direction would be to inquire about the *BAP1*-TPDS population’s motivations and barriers to sharing results and diagnosis with their family members. Evaluating the makeup of each family structure would further clarify who was available for this communication, as well as provide insight into participants’ reported motivations and barriers to sharing.

Qualitative studies, including structured interviews, could better capture participant perspectives and experiences. Prior research has shown interviews deepen understanding of family communication about genetic risk, coping with hereditary cancer syndromes, and motivations [18, 21, 30]. Others have found motivations such as moral obligation, health protection and decision-making, and identifying a familial cause [21, 30–32]. Challenges include loss of contact, concern about relatives’ reactions or distress, geographic distance, and complexity of the information [21, 26, 30–33]. Given the strong motivation to share in our *BAP1*-TPDS cohort, such studies could explore their unique challenges.

## Conclusion

Our study revealed that the *BAP1*-TPDS cohort is both motivated and interested in research participation; and is both knowledgeable and highly communicative about their diagnosis and genetic test results with all family members. Capturing the patient-reported outcomes regarding knowledge about their syndrome and experiences of the *BAP1*-TPDS population is imperative for better understanding where they may need additional education, resources, or support from the medical and research communities.

## Supplementary Information

Below is the link to the electronic supplementary material.

Supplementary Material 1

Supplementary Material 2

## Data Availability

Aggregate data that supports the findings of this study are available from the corresponding author upon reasonable request. Individual data will not be shared due to patients’ privacy and ethical restrictions.
