# Supplementary material for: Patient-derived outcome assessment of knowledge, communication, and management in those diagnosed with BAP1-tumor predisposition syndrome
Source: Fam Cancer. 2026 Mar 17;25(2):28. doi: 10.1007/s10689-026-00541-8 (PMC12996006; doi:10.1007/s10689-026-00541-8)
Supplement: Supplementary file 1 — Supplementary Material 1 [file 10689_2026_541_MOESM1_ESM.docx]

Table S-1. Additional Assessments.

| Question | Response Options | | | | | |
| --- | --- | --- | --- | --- | --- | --- |
| Are you involved in a support group for BAP1 tumor predisposition syndrome? | Yes | No | Prefer not to answer |  |  |  |
| If “yes”: What kind of support group are you involved in? Please provide details. | (open-ended response text box) |  |  |  |  |  |
| If there was a larger, non-social media, non-profit support group for BAP1 tumor predisposition syndrome, would you join? | Yes | No | Do not know |  |  |  |
| If “yes”: What would you want to see included in a BAP1 tumor predisposition syndrome support group (ex: information about BAP1, research updates and opportunities, connection to others, etc.)? | (open-ended response text box) |  |  |  |  |  |
| Have you ever had contact with a genetic counselor or genetics specialist about BAP1 tumor predisposition syndrome or your BAP1 result? | Yes | No | Do not know | Prefer not to answer |  |  |
| If “yes”: How long ago did you have contact with a genetic counselor or genetics specialist? | Within the last 6 months | Within 1 year | Within 2 years | More than 2 years ago | Do not remember | Prefer not to answer |
| Did you have to educate your healthcare provider about BAP1 (ex: cancer risks and/or screening recommendations)? | Yes | No | Do not know | Prefer not to answer |  |  |

How much have you discussed the following *BAP1* related risk topics with a health care provider? Please select what your level of discussion has been.

| **Topics discussed with physician** | **Not at all** | **Some** | **A lot** | **Do not remember** | **Prefer not to answer** |
| --- | --- | --- | --- | --- | --- |
| Risk of cancers for family members |  |  |  |  |  |
| Risk of developing skin cancer |  |  |  |  |  |
| Risk of developing eye cancer |  |  |  |  |  |
| Risk of developing kidney cancer |  |  |  |  |  |
| Risk of developing mesothelioma |  |  |  |  |  |
| Risk of developing other cancer (please specify): ______ (Open text box) |  |  |  |  |  |

How often do you have the following screenings?

| **Surveillance** | **I have never had this screening** | **Every 6 months** | **Yearly** | **Every 2 years** | **Do not know** | **Prefer not to answer** | **Other (explain)** |
| --- | --- | --- | --- | --- | --- | --- | --- |
| Physical Exam by primary care provider |  |  |  |  |  |  |  |
| Skin exam by dermatologist |  |  |  |  |  |  |  |
| Exam of the back of the eye after dilatation by ophthalmologist or optometrist |  |  |  |  |  |  |  |
| Photos of the back of the eye |  |  |  |  |  |  |  |
| Ultrasound of the abdomen |  |  |  |  |  |  |  |
| MRI of the abdomen |  |  |  |  |  |  |  |
| Other (Please specify): _____ |  |  |  |  |  |  |  |
