# Supplementary material for: Patient-derived outcome assessment of knowledge, communication, and management in those diagnosed with BAP1-tumor predisposition syndrome
Source: Fam Cancer. 2026 Mar 17;25(2):28. doi: 10.1007/s10689-026-00541-8 (PMC12996006; doi:10.1007/s10689-026-00541-8)
Supplement: Supplementary file 2 — Supplementary Material 2 [file 10689_2026_541_MOESM2_ESM.docx]

Table S-2. Participant and non-participant information.

|  | **Participant** | **Non-Participant** |
| --- | --- | --- |
| **Relationship** | | |
| Proband | 22 | 14 |
| Non-Proband | 20 | 21 |
| **History of Cancer Diagnosis** | | |
| Positive | 29 | 18 |
| Negative | 13 | 17 |
| **Proband and Negative Personal Cancer History** | 4 | 4 |
| **Type of Cancer/Tumor if Positive Cancer History^a^** | | |
| Adrenocortical cancer | 0 | 1 |
| Basal cell carcinoma | 8 | 9 |
| BIMTs | 4 | 2 |
| Bladder cancer | 2 | 0 |
| Breast cancer | 6 | 3 |
| Cholangiocarcinoma | 1 | 2 |
| Chronic lymphocytic leukemia | 1 | 0 |
| Colon cancer | 2 | 1 |
| Cutaneous melanoma | 5 | 5 |
| Liver cancer | 0 | 1 |
| Lung cancer | 2 | 0 |
| Malignant peripheral nerve sheath tumor | 1 | 0 |
| Meningioma | 3 | 2 |
| Mesothelioma | 2 | 2 |
| Neuroendocrine cancer | 1 | 0 |
| Oral cancer | 0 | 1 |
| Ovarian cancer | 1 | 0 |
| Prostate cancer | 1 | 0 |
| Renal cell carcinoma | 9 | 2 |
| Squamous cell carcinoma | 3 | 0 |
| Thyroid cancer | 0 | 1 |
| Ureteral cancer | 0 | 1 |
| Uveal melanoma | 6 | 0 |
| **History of BIMT and Negative Personal Cancer History** | | |
| Proband | 3 | 2 |
| Non-Proband | 0 | 0 |
| **Tested & Enrolled at OSU** | | |
| Yes | 7 | 4 |
| No | 35 | 31 |

^a^ Multiple types of cancer reported in an individual were counted separately.
